# Impaired Olfactory Associative Behavior of Honeybee Workers Due to Contamination of Imidacloprid in the Larval Stage

**DOI:** 10.1371/journal.pone.0049472

**Published:** 2012-11-14

**Authors:** En-Cheng Yang, Hui-Chun Chang, Wen-Yen Wu, Yu-Wen Chen

**Affiliations:** 1 Department of Entomology, National Taiwan University, Taipei, Taiwan; 2 Graduate Institute of Brain and Mind Sciences, National Taiwan University, Taipei, Taiwan; 3 Department of Animal Science, National Ilan University, Ilan, Taiwan; Université Paris 13, France

## Abstract

The residue of imidacloprid in the nectar and pollens of the plants is toxic not only to adult honeybees but also the larvae. Our understanding of the risk of imidacloprid to larvae of the honeybees is still in a very early stage. In this study, the capped-brood, pupation and eclosion rates of the honeybee larvae were recorded after treating them directly in the hive with different dosages of imidacloprid. The brood-capped rates of the larvae decreased significantly when the dosages increased from 24 to 8000 ng/larva. However, there were no significant effects of DMSO or 0.4 ng of imidacloprid per larva on the brood-capped, pupation and eclosion rates. Although the sublethal dosage of imidacloprid had no effect on the eclosion rate, we found that the olfactory associative behavior of the adult bees was impaired if they had been treated with 0.04 ng/larva imidacloprid in the larval stage. These results demonstrate that a sublethal dosage of imidacloprid given to the larvae affects the subsequent associative ability of the adult honeybee workers. Thus, a low dose of imidacloprid may affect the survival condition of the entire colony, even though the larvae survive to adulthood.

## Introduction

Honeybees play critical roles in agriculture and the global ecosystem by pollinating plants while at the same time producing bee products with a high economic value [1], [2], [3]. However, the rather recent phenomenon of colony collapse disorder (CCD) involving the sudden and massive disappearance of bee colonies around the world is worrisome [4]. This phenomenon manifests itself with the en masse disappearance of adult bees with only a few adult bee bodies being found around the beehives. The reason for this tends to be that these bees died while away from the hive, collecting pollen and nectar in the field, and were unable to navigate back home, thus leading to CCD. The severe loss of bee products and agricultural products caused by CCD [5], [6], [7] is of great concern to academics as well as to farmers. Recent studies show that RNA-induced acute paralysis virus, chronic paralysis virus, Kashmir bee virus (KBV), deformed wing virus (DWV), Israeli acute paralysis disease (IAPV), and disease caused by *Nosema ceranae* all contribute to CCD [8], [9], [10], [11]. However, the large scale use of systemic insecticides, such as imidacloprid and thiamethoxam, has also been considered as being a major contributing factor to CCD. Thiamethoxam has recently been demonstrated to induce homing failure that could potentially cause colony collapse as a result of a sublethal dosage as low as 1.34 ng/bee (i.e. an acute oral exposure to 20 µL of liquid containing 67 µg/L of the insecticide) [12]. An increasing number of studies imply that imidacloprid is also associated with colony disorder. Therefore, this study investigated the effect of imidacloprid on honeybees.

Imidacloprid is a neonicotinoid neurotoxic insecticide. Its major effect is acting on the nicotinic acetylcholine receptors (nAChR) of insects [13], [14], causing death of the nervous system due to hyper-excitation followed by paralysis [15], [16]. It has been considered an ideal insecticide because its toxicity is higher to insects than to mammals [17]. Imidacloprid is a systematic insecticide with contact toxicity and gastric toxicity. It can be applied either by foliar spray application, granular spray application, irrigation or seed treatment to protect plants from pest invasions [18]. In 1994, an event of obvious weakening of bee colonies, and a reduction in bee products was reported after the suspected consumption of sunflower nectar by the bees resulted in abnormal honeybee behavior to a point of the bees failing to return home [5], [6]. This event is likely attributed to the fact that sunflower Gaucho® seeds were used in that area of France. Gaucho® seed is a seed treated with imidacloprid. Imidacloprid spreads to every part of the plant through the vascular system (i.e. xylem) and kills insects that feed from the plant. The sunflower Gaucho® seeds event increased the awareness of the important influence the use of imidacloprid has on honeybees, and resulted in an increased interest in studying the median lethal dosage (LD_50_) and median lethal concentration (LC_50_) of imidacloprid on honeybees [5], [6], [18], [19], [20], [21], [22], [23]. Although LD_50_ and LC_50_ are the prime toxicity indicators for insecticides, the sublethal dosage of imidacloprid on honeybees needs to be considered as well. Previous studies have shown that after feeding the honeybees imidacloprid, their activity level decreased at an imidacloprid concentration of 100 µg/kg [24], and in addition their collection activities changed at a concentration above 6–100 µg/kg [25], [26], [27], [28], [29], [30] while their olfactory learning and memory abilities were impaired at a concentration above 12–24 µg/kg [22], [27]. The bees' medium-term olfactory memory was impaired with an imidacloprid dose of 12 ng/bee [31]. Recently, Eiri and Nieh reported that the ingestion of imidacloprid of 0.21 ng/bee could increase the threshold of sucrose concentration that a forager would accept and reduce waggle dancing at different time scale which consequently diminished the fitness of the bee colony [32].

Honeybee foragers collect food outside the hives and thus have frequent contact with plants that are contaminated with imidacloprid [33], and thus they bring honey and pollen containing imidacloprid back to their hives for storage [34], [35], [36]. Nurse bees then feed the imidacloprid-containing honey and pollen to the bee larvae, which could possibly affect their development as a result of accumulating the influences of imidacloprid during their growth period. However, previous studies failed to demonstrate a reduced honeybee mortality rate, collection activities, beeswax production, larvae capped-brood rate, colony activities, homing rate, and pollen carrying rate after feeding imidacloprid to honeybees [6], [37]. It was therefore concluded that a low concentration of imidacloprid does not affect the bee colony and the larvae capped-brood rate. In the natural environment, imidacloprid concentration is less than 10 µg/kg in the soil, nectar and pollen [5], [6], [31], [34], [38], [39], [40], [41]. Nevertheless, this does not exclude the possibility of accumulative intoxication through the repetitive consumption of honey and pollen containing only a low concentration of imidacloprid. In addition to the flowering products of a plant, the resin sources for propolis are assumed to transfer this systemic insecticide into the bee colony as well [42]. The relatively high residue of imidacloprid was observed in the honeycomb and propolis of depopulated beehives [42], indicating that the insecticide could be accumulated in these materials, resulting in the larvae of the colonies being continuously exposed to the contamination before the hives were depopulated. However, previous studies focused on the investigation of adult bees and bee colonies rather than on larvae. Therefore, our study focused on providing honeybee larvae with various doses of imidacloprid and to investigate and observe if the larvae, capped-brood, pupation and eclosion rates as well as the olfactory associative behavior change after having been exposed to imidacloprid.

## Materials and Methods

### 1. Source of the test insects

*Apis mellifera* L. served as the test insect for this study. Honeybee colonies were raised in the apiaries of National Ilan University. Each test colony had a population working on about 9 frames of honeycomb in a Langstroth hive, and the test population included a queen bee spawning normally. The hives were checked every week to ensure that the normal function of the honeybee colony was being maintained.

### 2. Preparation of chemicals

Imidacloprid (95%, TG, Bayer Cropscience AG, Monheim am Rhein, Germany) is in powder form, thus it has to be dissolved in an organic solvent such as dimethylsulfoxide (DMSO) or acetone. We did not examine if there were any negative influences on the honeybees from the organic solvents because the amount of organic solvent was trivial in the end test solution (0.1 or 1%, v/v) [19], [20], [22], [27], [31], [37]. Previous studies showed that the feeding behavior of honeybees is not influenced by 0.1% DMSO treatment; however, the feeding activity of honeybees reduces significantly with acetone treatment [30]. We therefore chose DMSO (MP Biomedicals LLC., Solon, OH, USA) as the solvent for imidacloprid.

The imidacloprid stock solutions were prepared with concentrations of 8 and 200 g/L (imidacloprid in DMSO), respectively. Test solutions of imidacloprid were freshly prepared just prior to each application. The intermediate-concentration solutions of 100 and 6000 mg/L were prepared in advance by diluting the 8 g/L stock solution with DMSO. Then 2 µL of each of the above prepared intermediate-concentration solutions was added into 1998 µL of distilled deionized water (DDW), resulting in test solutions with imidacloprid concentrations of 0.1 and 6 mg/L. These test solutions contained 0.1% (v/v) DMSO. Another set of imidacloprid test solutions with higher concentration were prepared from the 200 g/L stock solution by adding 0.5, 5, 10, 15 and 20 µL of this stock solution to 1999.5, 1995, 1990, 1985 and 1980 µL of DDW respectively, resulting in an imidacloprid concentration of 50, 500, 1000, 1500 and 2000 mg/L with a DMSO concentration ≤1% in the imidacloprid test solution (the DMSO concentrations were 0.025, 0.25, 0.5, 0.75 and 1% respectively). In addition, a set of imidacloprid test solutions with sublethal concentration (according to the result of this study) were prepared for testing the effect of imidacloprid on olfactory association. The intermediate-concentration solutions of 0.01, 0.1, 1 and 10 mg/L were prepared by diluting the 8 mg/mL stock solution with DMSO. Fresh test solutions of imidacloprid were prepared by adding 20 µL of each of the above prepared intermediate-concentration solutions into 1980 µL of DDW respectively, resulting in imidacloprid concentrations of 0.1, 1, 10 and 100 µg/L with an uniform DMSO concentration of 1% (v/v) in the imidacloprid test solution.

### 3. Testing the effect of imidacloprid on larval survival

Four honeybee colonies were selected. The queens were restricted to depositing their eggs in empty frames of honeycomb using vertical and horizontal queen excluders for 24 hours in the test hives. The frames with eggs were left inside the test hives and checked daily. On the 3^rd^ day, the frames were removed and transparent slides were placed on the honeycombs to mark the relative locations of the cells occupied by 1-day-old (emerged within 24 hours) larvae with brood food. More than three hundred cells were marked in each colony. The larvae in the cells were divided into 10 groups, each consisting of 30 to 40 larvae, and each marked with a different color on the transparent slide. The larvae from 7 of these 10 groups were treated with different doses of imidacloprid by adding 1 µL of imidacloprid test solution each at the respective concentrations of 0.1, 6, 50, 500, 1000, 1500 and 2000 mg/L into their cells. The larvae from the other 3 groups were set as control groups in order to evaluate the effect of the solvents. For the control groups, the larvae were treated with 1 µL each of DDW and 0.1 and 1% (v/v) DMSO solutions respectively using the same procedures of the imidacloprid treatment groups. After the drug application, the marked transparent slides were removed and kept as a reference for the applications of the following days. The frames of honeycomb were then put back into the respective colony, and the larvae were allowed to be reared inside the colonies. The drug application was conducted once a day for 4 consecutive days (from 1 to 4 days old), and the total doses of imidacloprid added into the nest cells were 0.4, 24, 200, 2000, 4000, 6000 and 8000 ng/larva (nominal doses) respectively. Chemicals can be absorbed by the larvae orally or by contact. The capped-brood rate was calculated starting from day 7. On day 15, the pupae were removed from their colonies and placed on 24-hole plates to calculate the pupation rate. The pupae were later placed in a dark incubator at 34°C and 70% relative humidity without other bees for eclosion rate observation.

### 4. Treatment for testing the effect of imidacloprid after eclosion

An additional three honeybee colonies were selected for testing the effect of imidacloprid at a sublethal dosage on the contaminated larvae in adulthood. Seven groups of 100 one-day-old larvae each were obtained by the same procedures as described above. The other corresponding procedures of adding 1 µL of DDW, solution of 1% DMSO and solutions of 0.1, 1, 10 and 100 µg/L imidacloprid respectively once a day to each cell of the larvae among the 6 groups for 4 consecutive days (from 1 to 4 days old) were performed as well. The total amounts of imidacloprid added over 4 days were 0, 0, 0.0004, 0.004, 0.04 and 0.4 ng/larva (nominal doses) respectively. A group of 100 larvae was left intact as a control. On day 15, the pupae were removed from the colonies and placed on 24-hole plates and then transferred into the dark incubator under the conditions described above. The eclosion rate was about 90% for each group. Color labels were affixed to the dorsum of the thorax of the honeybees after eclosion so as to differentiate the honeybees of each group. After having been color-labeled, the honeybees were released into their original colony. The color-labeled honeybees were randomly selected to test their olfactory associative behavior 15 days after eclosion. Although worker bees turn into foragers 20 days after eclosion [43], our pretest showed that sampling difficulties occurred if the olfactory association was to be tested on day 20. This difficulty was possibly due to the honeybees' homing failure similar to the phenomenon that was demonstrated in the adulthood of a forager when subjected to acute contamination by neonicotinoids [12]. It has been shown that worker bees can associate on day 15 after eclosion [44]. Therefore, we choose the color-labeled honeybees on day 15 after eclosion to test the olfactory associative behavior of the worker bees.

### 5. Proboscis extension reflex (PER) test

Conventional conditioning of the proboscis extension reflex (PER), as shown in many previous studies, was used to test the honeybees' associative ability [22], [45], [46], [47], [48], [49]. We applied the principles of classical conditioning [50] with odor as the conditioned stimulus (CS) and sugar water as the unconditioned stimulus (US) to test the olfactory associative behavior of the honeybees by observing their PER. Honeybees were placed in a dark incubator at 25°C and 40% relative humidity where they were starved for 4 hours before the test. After the 3^rd^ hour, the honeybees were anesthetized by low temperature by being placed in a 4°C ice bucket for 5–10 minutes. After anesthesia, honeybees were fixed at 1000 µL pipette tips by beeswax/resin mixture, left there for 1 hour until their physiological conditions recovered. Cotton swabs dipped with 50% (w/v) sucrose solution were applied over the honeybees' antenna (precautions were taken not to let the honeybees take in any sugar water). Honeybees with a normal PER were tested for their olfactory associative behavior. Those that could not produce a PER were eliminated. Groups of approximately thirty honeybees per group were tested for their olfactory associative behavior after various treatments.

When training the bee to associate, the bees were offered 50% sucrose solution for 3 seconds. Then the odorous stimulation was applied for 6 seconds by placing the bees 1 cm away from the blow hole of a pneumatic PicoPump (PV380, World Precision Instruments, Inc., Sarasota, FL, USA) connected to a bottle filled with citral (≥96%, FG, W230308, Sigma-Aldrich, Inc., St. Louis, MO, USA). At the 3^rd^ second the bees were simultaneously offered sugar water while continuing to experience the odor.

The test for the PER by providing odorous stimulation only was conducted to observe if the honeybees associated this stimulation with sugar water. Each honeybee went through 4 associative tests (Conditioning 1–4) and each test was held 20 minutes apart. The successful rate of the PER response rate was calculated according to Ray and Ferneyhough [44]:
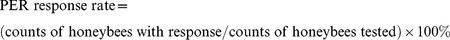



### 6. Statistical analysis and calculation of medial lethal dose (LD_50_)

SPSS software (IBM Corp., Armonk, NY, USA) was used for the statistical analysis in this study. The effects of organic solvents and imidacloprid on the larvae capped-brood rate, pupation rate, eclosion rate as well as honeybee's olfactory associative behavior after eclosion were analyzed by the two-tailed Kruskal-Wallis H tests, because the data were not distributed normally, and a two-tailed Mann-Whitney U test with the Dunn-Šidák correction at the 95% confidence level was performed as a post-hoc test. In this study each data was shown as the mean ± standard deviation. The CalcuSyn software (Biosoft, Ferguson, MO, USA) was used to calculate the LD_50_ of imidacloprid for the honeybee larvae capped-brood rate.

## Results

### 1. Influence of DMSO on larvae capped-brood rate, pupation rate and eclosion rate

Larvae capped-brood rates were 100±0, 98.75±2.50 and 91.04±9.36% for larvae treated with DDW, 0.1 and 1% DMSO, respectively. The analysis showed there was a variation among the larvae capped-brood rates (two-tailed Kruskal-Wallis H test, *χ* ^2^ = 18.646, *df* = 2, *P*<0.001). The capped-brood rate of the group of 1% DMSO was slightly lower than the rates of the groups of DDW (two-tailed Mann-Whitney U test with Dunn-Šidák correction, *U* = 10320, adjusted *P*<0.001) and 0.1% DMSO (two-tailed Mann-Whitney U test with Dunn-Šidák correction, *U* = 10137.5, adjusted *P* = 0.018<0.05). Nevertheless, the pupation rates were 96.25±3.23, 97.50±3.54 and 91.04±9.36%, showing no significant difference among the three treatment groups (two-tailed Kruskal-Wallis H test, *χ* ^2^ = 5.061, *df* = 2, *P* = 0.08). Eclosion rates were 89.38±6.57, 89.82±10.10 and 89.17±7.26%, showing no significant difference (two-tailed Kruskal-Wallis H test, *χ* ^2^ = 0.031, *df* = 2, *P* = 0.984) as well. We summarized these results, and concluded that the DMSO applied in this study had an ignorable influence on the development of the larvae.

### 2. Influence of imidacloprid on the larvae capped-brood rate

**Figure 1:**
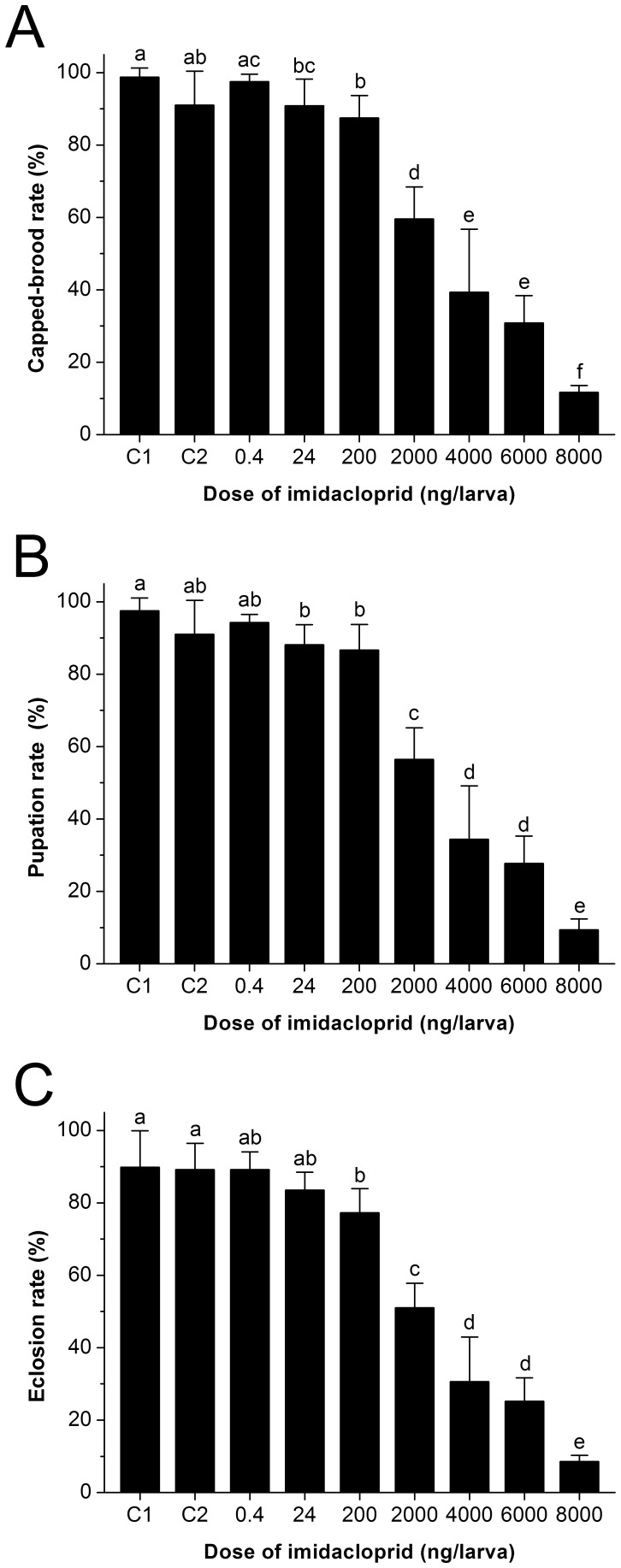
Lethal effect of imidacloprid on the honeybee larvae. The subfigures show the effects of imidacloprid on the capped-brood rate (A), pupation rate (B) and the eclosion rate (C) of honeybee larvae under field conditions (Tested larvae were obtained from 4 colonies, *N*_0.1% DMSO_ = 40+40+35+40, *N*_1% DMSO_ = 30+40+40+30, *N*_0.4 ng_ = 40+40+35+40, *N*_24 ng_ = 30+30+40+30, *N*_200 ng_ = 30+40+40+30, *N*_2000 ng_ = 40+40+30+30, *N*_4000 ng_ = 40+40+30+30, *N*_6000 ng_ = 40+40+30+30, *N*_8000 ng_ = 30+40+30+30 larvae). The doses of imidacloprid treatments are 0.4, 24, 200, 2000, 4000, 6000 and 8000 ng/larva. C1 and C2 are the control groups (0.1 and 1% DMSO). In each subfigure, any two effects of the experimental treatments without any same letter above the columns are significantly different (two-tailed Kruskal-Wallis H test, *P*<0.001, compared by two-tailed Mann-Whitney U tests with Dunn-Šidák correction at the 95% confidence level).

Larvae capped-brood rates were 97.50±2.04, 90.83±7.39 and 87.50±6.16% for 0.4, 24 and 200 ng/larva imidacloprid treatment, while the rates were 98.75±2.50 and 91.04±9.36% for 0.1 and 1% DMSO treatment (control group). With the dose raised to 2000, 4000, 6000 and 8000 ng/larva, most larvae were removed by the nurse bees by day 2 or day 3 with larvae capped-brood rates of 59.58±8.83, 39.38±17.37, 30.83±7.55 and 11.67±1.92% respectively. Larvae capped-brood rates were significantly different among the experimental treatments (two-tailed Kruskal-Wallis H test, *χ* ^2^ = 560.317, *df* = 8, *P*<0.001). There was no significant difference between the control group of 0.1% DMSO and the group that received low dose imidacloprid treatment at a dose of 0.4 ng/larva (two-tailed Mann-Whitney U test with Dunn-Šidák correction, *U* = 11857.5, adjusted *P* = 0.991), but the groups that received a higher dose above or equal to 24 ng/larva differed significantly from the control group (two-tailed Mann-Whitney U tests with Dunn-Šidák corrections, adjusted *P*<0.05) (Figure 1A). The LD_50_ of imidacloprid is about 1400 ng/larva as analyzed by the CalcuSyn software.

### 3. Influence of imidacloprid on the pupation rate

Pupation rates were 94.29±2.24, 88.13±5.54 and 86.67±7.07% with 0.4, 24 and 200 ng/larva imidacloprid while the rates were 97.50±3.54 and 91.04±9.36% for the control group with 0.1 and 1% DMSO. With the imidacloprid dose raised to 2000, 4000, 6000 and 8000 ng/larva, the pupation rates were 56.46±8.72, 34.38±14.77, 27.71±7.56 and 9.38±2.99% respectively. Pupataion rates were significantly different among the experimental treatments (two-tailed Kruskal-Wallis H test, *χ* ^2^ = 565.454, *df* = 8, *P*<0.001). The pupation rates were not different between the control group of 0.1% DMSO and the group that received low dose imidacloprid treatment at a dose of 0.4 ng/larva (two-tailed Mann-Whitney U test with Dunn-Šidák correction, *U* = 11625, adjusted *P* = 0.785), but the groups that received a higher dose above or equal to 24 ng/larva were significantly different from the control group (two-tailed Mann-Whitney U tests with Dunn-Šidák corrections, adjusted *P*<0.05) (Figure 1B).

### 4. Influence of imidacloprid on the eclosion rate

Eclosion rates were 89.20±4.90, 83.54±4.92 and 77.29±6.64% with 0.4, 24 and 200 ng/larva imidacloprid while the rates were 89.82±10.10 and 89.17±7.26% for the control group. With imidacloprid dose raised to 2000, 4000, 6000 and 8000 ng/larva, the eclosion rates were 51.04±6.78, 30.63±12.31, 25.21±6.47 and 8.54±1.72%, respectively. Eclosion rates were significantly different among the experimental treatments (two-tailed Kruskal-Wallis H test, *χ* ^2^ = 486.874, *df* = 8, *P*<0.001). The eclosion rates were not different between the control group of 0.1% DMSO and the groups that received low dose imidacloprid treatments at a dose of 0.4 ng/larva (two-tailed Mann-Whitney U test with Dunn-Šidák correction, *U* = 11935, adjusted *P* = 1) and 24 ng/larva (two-tailed Mann-Whitney U test with Dunn-Šidák correction, *U* = 9487.5, adjusted *P* = 0.756), respectively, but the groups that received a higher dose above or equal to 2000 ng/larva differed significantly from the control group (two-tailed Mann-Whitney U tests with Dunn-Šidák corrections, adjusted *P*<0.05)(Figure 1C).

### 5. Influence of DMSO on the olfactory associative behavior of honeybees

**Figure 2:**
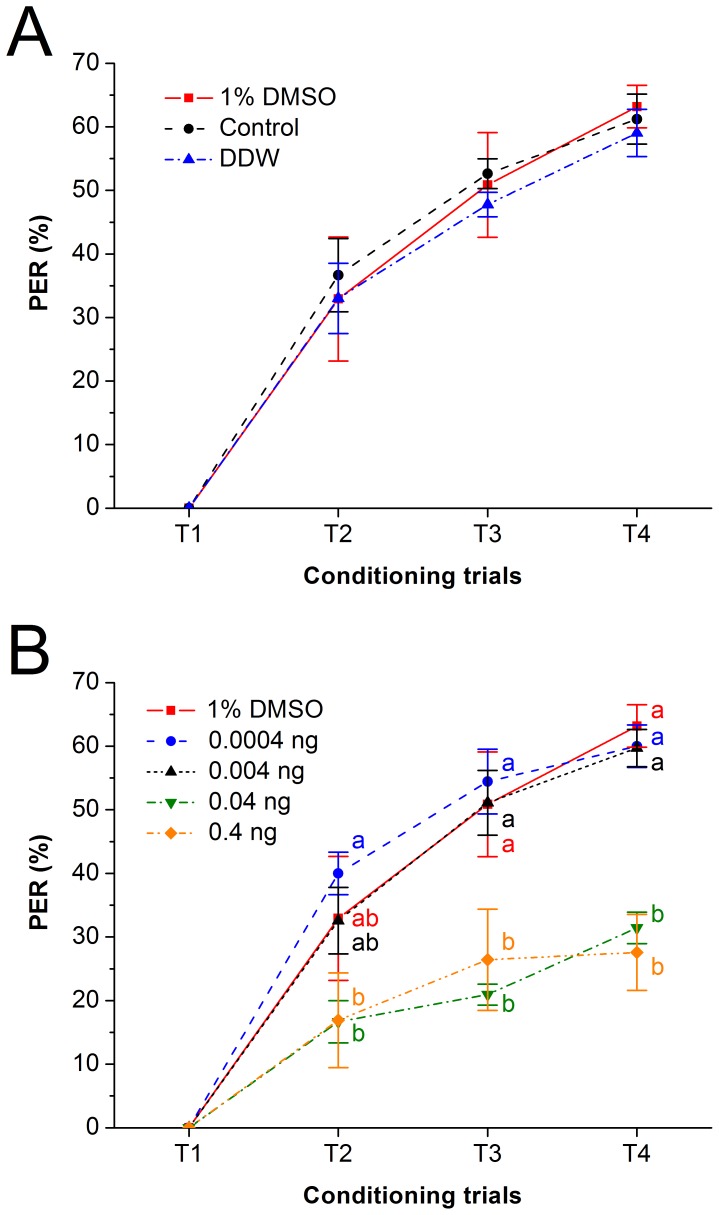
Impaired olfactory associative behavior in adulthood caused by the larval contamination of imidacloprid. The subfigures show the effect of DMSO (A) and imidacloprid (B) on the olfactory associative behavior of the honeybee (Tested honeybees were obtained from 3 colonies, *N*_1% DMSO_ = 35+25+30, *N*_Control_ = 33+30+30, *N*_DDW_ = 30+30+28, *N*_0.0004 ng_ = 30+30+30, *N*_0.004 ng_ = 32+30+30, *N*_0.04 ng_ = 30+30+35, *N*_0.4 ng_ = 26+29+32 bees). T1, T2, T3 and T4 are conditioning trials 1–4, respectively. In figure 2B, any two effects of the experimental treatments among T2, T3 or T4 without any same letter beside the data points are significantly different (two-tailed Kruskal-Wallis H test, *P*≤0.001, compared by two-tailed Mann-Whitney U tests with Dunn-Šidák correction at the 95% confidence level).

The olfactory associative behavior of the larvae treated with DDW and 1% DMSO 15 days after eclosion is shown in Figure 2A. For the DDW treated group, the PER rate was 0% for conditioning trial 1 (T1), 33.02±5.52% for conditioning trial 2 (T2), 47.78±1.92% for conditioning trial 3 (T3) and 59.05±3.72% for conditioning trial 4 (T4). For the control group, the PER rate was 0% for T1, 36.67±5.77% for T2, 52.63±2.35% for T3 and 61.21±3.94% for T4. For the 1% DMSO treated group, PER rate was 0% for T1, 32.92±9.75% for T2, 50.86±8.24% for T3 and 63.17±3.34% for T4. The PER responses of these groups showed no significant difference (two-tailed Kruskal-Wallis H tests, T2: *χ* ^2^ = 0.317, *df* = 2, *P* = 0.853; T3: *χ* ^2^ = 0.46, *df* = 2, *P* = 0.794; T4: *χ* ^2^ = 0.336, *df* = 2, *P* = 0.845). These results indicate that the influence from 1% DMSO on the olfactory associative behavior after eclosion can be ignored.

### 6. Influence of imidacloprid on the olfactory associative behavior of honeybees

The curve of larvae treated with 0.0004–0.4 ng/larva imidacloprid 15 days after eclosion is shown in Figure 2B. For larvae treated with a low dose of 0.0004 ng imidacloprid, the response rates were 0, 40.00±3.33, 54.44±5.09 and 60.00±3.33% for T1–T4. With the dose raised to 0.004 ng, the response rates were 0, 32.57±5.24, 51.11±5.09 and 59.72±2.93% for T1–T4. With the relatively high dose of 0.04 ng, the response rates were 0, 16.67±3.33, 20.95±1.65 and 31.43±2.47% for T1–T4. With 0.4 ng imidacloprid treatment, the response rates were 0, 16.90±7.45, 26.42±7.95 and 27.57±5.96% for T1–T4. Results of the two-tailed Kruskal-Wallis H tests revealed that there were significant differences of PER rates among 1% DMSO and imidacloprid treated groups in T2 (*χ* ^2^ = 18.348, *df* = 4, *P* = 0.001<0.05), T3 (*χ* ^2^ = 35.976, *df* = 4, *P*<0.001) and T4 (*χ* ^2^ = 43.413, *df* = 4, *P*<0.001), respectively. The bees belonging to the 1% DMSO and 0.0004 and 0.004 ng imidacloprid treated groups showed better olfactory associative ability than the bees treated with 0.04 and 0.4 ng imidacloprid in T3 and T4 (two-tailed Mann-Whitney U tests with Dunn-Šidák corrections, adjusted *P*<0.05), and the bees belonging to the 0.0004 ng imidacloprid treated group showed better olfactory associative ability than the bees treated with 0.04 and 0.4 ng imidacloprid in T2 (two-tailed Mann-Whitney U tests with Dunn-Šidák corrections, adjusted *P*<0.05)(Figure 2B).

## Discussion

In our field test with larvae, the larvae were most of the time raised by the colony except when they were treated with imidacloprid by the experimenters. This method is as close as possible to the natural condition of the honeybee colony, which the larval survival was affected both by its development and the hygienic behavior, which ejected unhealthy larva, of the nurse bee. Thus, we applied it to evaluate the effects of exposing the larvae to imidacloprid contamination in the field. Our results showed that the capped-brood, pupation and eclosion rates as well as the subsequent olfactory associative ability in adulthood were affected by imidacloprid. The relative high survival rates of control groups and low dosage (below and equal 200 ng/larva) treatment groups against the dosage-related decline of the survival rates of high dosage (above and equal 2000 ng/larva) treatment groups indicates the cross contamination of treated imidacloprid was not occurred in this study. In addition, the high dosage of imidacloprid would knock down and kill a nurse bee (NOEC of the knockdown effect = 0.94 ng/bee [51], [52], LD_50_ = 3–81 ng/bee [5], [6], [18], [19], [20], [21], [22], [23]) and would result in larval deaths due to starvation. However, this was not observed in the high dose (above and equal 2000 ng/larva) treatment groups of this study, indicates the contaminated brood food should not be removed by nurse bees when the larva alive. Nevertheless, a damage of nursing task may not be excluded when the nursing behavior was not examined in this study.

Imidacloprid is a systematic insecticide and their residue could be found in the plant tissue and in the soil after spraying or seed-coating treatment. Previous studies have shown that the concentration of imidacloprid residue is below 10 µg/kg in the soil, nectar and pollen in an argo-environment [5], [6], [34], [38], [39], [40], [41], [53]. Because a honeybee larva can consume 160 µL of brood food before its pupation [54], it is quite possible that honeybee larvae are affected by the environmental residue of imidacloprid. In this study, we tested the dose at 0.0004, 0.004, 0.04 and 0.4 ng/larva, which corresponds to expose the larvae to an imidacloprid concentration of approximately 0.0025, 0.025, 0.25 and 2.5 µg/L respectively, which represents the level that is very likely present in an argo-environment. The result revealed that the adult olfactory associative behavior was impaired if the brood-food was contaminated by the imidacloprid treatment at a concentration of 0.25 µg/L or more. This is strong evidence and indicates that a honeybee larva could remain exposed to the residual imidacloprid in an agro-environment. Because honeybee larvae do not consume raw nectar or pollen, we presumed that they were protected from the contamination of a bee colony, or at least that they were protected by the repellent effect of imidacloprid on the forager [25] and the detoxification abilities of a nectar-collecting forager and a larva food-preparing nurse bee [55]. Nevertheless, because the detoxification gene is deficient in a honeybee [56], this protection may break down under the synergy of other stresses, such as malnutrition, disease and the intoxication by insecticides of adult workers, and result in colony disorder.

According to our results, the larva capped-brood rate was not influenced by imidacloprid below 0.4 ng/larva. However, the larvae capped-brood rate was significantly reduced when the imidacloprid dose was increased to 24 ng/larva and more (Figure 1A). The calculated imidacloprid LD_50_ is about 1400 ng/larva. Previous studies show that imidacloprid LD_50_ is 3–81 ng/bee for adult bees [5], [6], [18], [19], [20], [21], [22], [23]. Comparing our results with previous studies, it is obvious that larvae have a higher tolerance to imidacloprid than adult bees. Imidacloprid acts mainly on a specific nAChR rather than on all nAChRs presented in a victim [31]. A honeybee possesses as many as 11 members of insect nAChR subunits [57], and these subunits are expressed differently in the different stages of honeybee development [58], [59]. Furthermore, many structures are absent in the early stage of a larva such as the Kenyon cells in the mushroom bodies (MBs) [60], [61] which are the primary target of nAChR for imidacloprid [62], [63], [64]. The high tolerance for imidacloprid therefore may be related to the fact that the larvae lack nAChR, which has a high agonistic affinity for imidacloprid in adults. In addition, it has been demonstrated that feeding laboratory reared larva with artificial food contaminated with imidacloprid at a rate as high as 400 mg/kg will induce apoptotic cell death in the tissue of a larva's midgut [65]. We therefore assume that the lethal effect on larvae treated with a high dose of imidacloprid may be attributed to an induced apoptosis rather than neural toxicity.

The understanding of the sublethal effect of imidacloprid on honeybee larvae is relatively poorer than what has been reported for the adult bee. Decourtye et al. reported a delayed development of honeybee larvae that were fed food contaminated with imidacloprid at 5 µg/kg [23], [66]. This delayed-development effect was also observed in the honeybee larvae reared in honeycomb that contained residue of insecticides, including imidacloprid at 45 µg/kg [67]. A similar effect on larvae exposed to imidacloprid at 30–300 µg/kg was confirmed as well for another pollinator, *Osmia lignaria* [68]. In the present study, we report an effect that can be induced by a tiny dosage, as low as 0.04 ng/larva (or about 0.25 µg/L of the exposed concentration for 4 days), to impair the olfactory associative behavior in adulthood. Imidacloprid has a metabolic half-life ranging between 4.5–5 hour [69] in the adult honeybee, but the metabolites of imidacloprid, 5-hydroxy-imidacloprid, olefin and 4,5-dihydroxy-imidacloprid remain toxic to the honeybee [20], [21], [22], [69], [70], [71]. Since the metabolism of the larvae remains unclear, we presumed that the imidacloprid and its metabolites could accumulate in the larvae and have a negative effect on the larvae's development. The imidacloprid's primary target, nAChR, could be linked to the development of the brain and the neural plasticity [72], [73]. In an adult honeybee's brain, the nAChRs are mostly distributed among the MBs, antennal lobes (ALs), antennal nerves, visual ganglions, central body and the suboesophageal ganglion [74], [75], [76]. Functional cytochrome oxidase histochemical studies have shown that the application of imidacloprid could significantly increase the neural metabolic activity among the lip and basal ring of the MBs rather than among the α-lobe of the MBs and ALs [31], [77]. When honeybees are treated with nicotinic antagonist—mecamylamine, muscarinic and imidacloprid, then these chemicals bind with the nAChR of the MBs in the brain causing an impaired memory and learning ability [78], [79]. The learning ability of honeybees is significantly reduced when they are fed a sucrose solution containing imidacloprid at 12, 24, 48, or 96 µg/kg [22]. In addition, the mid-term memory of honeybees is impaired when they are fed a dose of imidacloprid of 12 ng/bee [31]. Furthermore, the ingestion of imidacloprid at 0.21 ng/bee could increase the threshold of acceptable sucrose concentration of a forager after 1 hour and reduce waggle dancing 24 hours later [32]. Due to the fact that the symptoms of these above-mentioned acute intoxications of adults and the impairment of the olfactory associative behavior observed in this study seem to be related, it also is possible that the brain functions affected in these events may be connected with each other. We expect that the accumulated imidacloprid and its metabolites may interfere with the development of the functional regions of the brain such as in the MBs, ALs as well as the antennal nerves.

Besides the effect on individual larva, a sublethal dosage of imidacloprid (contaminant concentration at 0.7–10 µg/kg) also contributes to reducing the fertility of the queen and the colony growth of bumble bee colonies [80], [81]. However, due to the experimental period was as short as the lifespan of a worker, it is very likely that the sublethal effect observed in this study is an original factor that affects the colony yet does not show the response from the colony.

In summary, this study demonstrated that the honeybee larvae are more tolerant to imidacloprid than the adult bees, but that their development, at least that of the MBs, ALs and antennal nerves may be very easily interfered with by imidacloprid contamination. Honeybees depend on the MBs and ALs in the brain to learn and memorize food location as well as their homing routes when they are out collecting [74], [78], [79], [82], [83], [84], [85]. Our results infer that although imidacloprid does not kill the larvae, when these honeybees with both learning and memory impairments go out collecting, it is highly likely that they cannot learn and memorize food locations and homing routes and that therefore they fail to return to their hives, causing a reduction of bee products and getting even worse to induce CCD. Because honeybee larvae could be affected by a contamination of imidacloprid contamination as low as 0.04 ng/larva, neonicotinoid insecticides should be applied very carefully.
